# A Case of Pediatric Supracondylar Humerus Fracture Requiring Vascular Reconstruction for Brachial Artery Injury: The Importance of Rapid Vascular Assessment

**DOI:** 10.7759/cureus.91503

**Published:** 2025-09-02

**Authors:** Yasuaki Maeda, Shinichiro Okimura, Yuko Narita, Akira Katsumata, Atsushi Teramoto

**Affiliations:** 1 Department of Orthopaedic Surgery, Obihiro Kyokai Hospital, Obihiro, JPN; 2 Department of Orthopaedic Surgery, Sapporo Medical University, School of Medicine, Sapporo, JPN

**Keywords:** brachial arterial injury, pediatric supracondylar humerus fracture, pulseless hand, surgical exploration, vascular reconstruction

## Abstract

Supracondylar humeral fractures are the most commonly observed pediatric fractures in clinical practice. Associated brachial artery injuries are rare complications; however, published case reports documenting the need for vascular reconstruction are limited. An eight-year-old boy presented with pain in the left elbow after he fell. Radiograph revealed a severely displaced supracondylar humeral fracture. Initially, the radial pulse was absent; nonetheless, the fingers remained perfused. However, the rapid onset of digital pallor required urgent open reduction and vascular reconstruction. Bone union was achieved within seven weeks postoperatively, with restoration of symmetrical radial pulses. In this case, the risk of rapid vascular deterioration in pediatric supracondylar humeral fractures was emphasized. Frequent vascular assessments are essential, as vigilant monitoring and prompt intervention can mitigate complications, prevent long-term sequelae, and ensure better patient outcomes.

## Introduction

Supracondylar humeral fractures are the most common elbow fractures in children [1] and are frequently encountered in clinical practice. Additionally, brachial artery injury is not uncommon in association with these fractures, with reported incidence rates ranging from approximately 4% to 23% [2,3]. In a six-year study of 116 pediatric supracondylar humeral fractures, only one case required vascular reconstruction owing to persistent ischemia after reduction [4]. Herein, we report a case of a pediatric supracondylar humeral fracture where vascular status rapidly deteriorated shortly after hospital arrival, necessitating urgent vascular reconstruction. Through this case, we aim to highlight strategies for the early detection of vascular compromise and prevention of complications in these fractures.

## Case presentation

**Figure 1:**
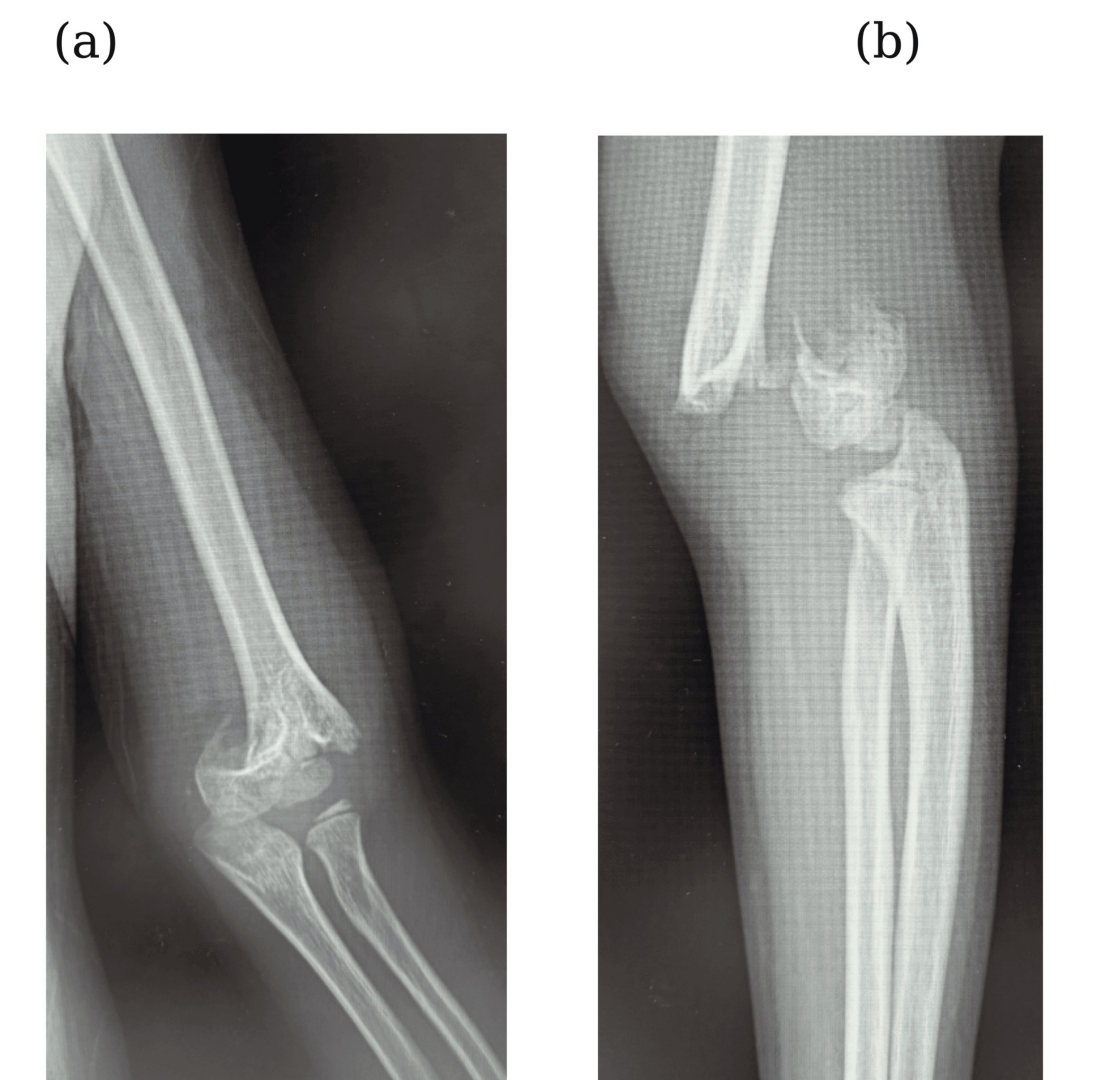
Initial plain radiograph in (a) frontal view and (b) lateral view taken at the first hospital visit.

An eight-year-old boy was presented to our emergency department with left elbow pain after falling from a 50 cm-high chair, landing on his outstretched left hand. Initial examination, two hours post-injury, revealed a deformed left upper arm and an absent radial pulse. However, the capillary refill time (CRT) was less than two seconds, and no pallor or coldness of the fingers was observed. The radial artery pulse was not palpable, but peripheral perfusion was preserved, indicating a pulseless pink hand (PPH) condition. Neurological deficits were not observed. A Gartland type III supracondylar fracture of the left humerus was confirmed from radiographs (Fig. 1a, b). At the time of operating room admission, approximately three hours post-injury, the patient’s fingers appeared pale and CRT was prolonged, measuring more than three seconds, suggesting vascular compromise due to compression or strangulation of the vessel at the fracture site. Considering the risk that closed reduction could exacerbate the vascular entrapment or compression, we opted for immediate open reduction and vascular exploration through an anterior approach to the elbow.

**Figure 2:**
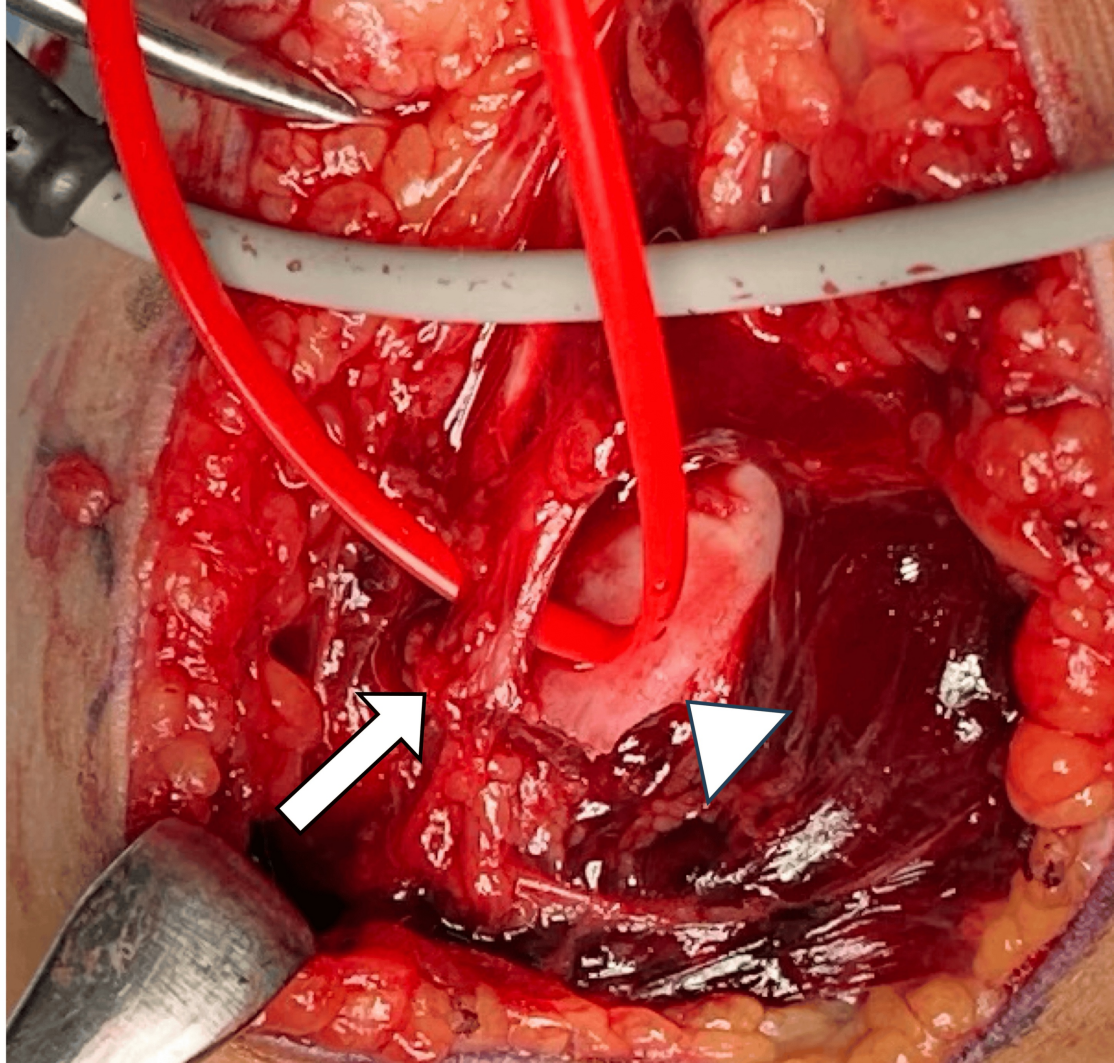
The displaced humerus fragment (arrowhead) compressing the brachial artery (arrow).

**Figure 3:**
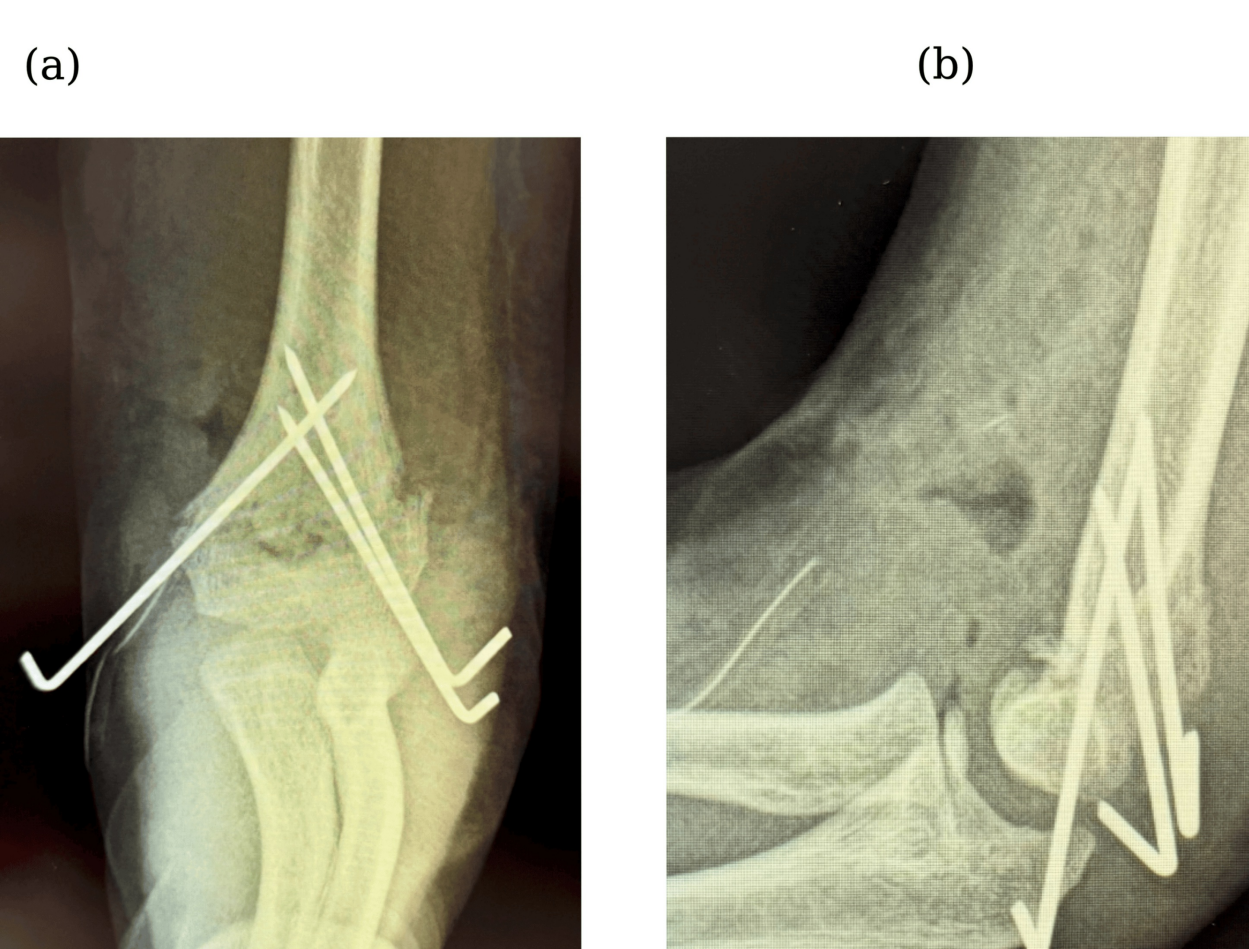
Plain radiograph in (a) frontal view and (b) lateral view taken postoperatively.

**Figure 4:**
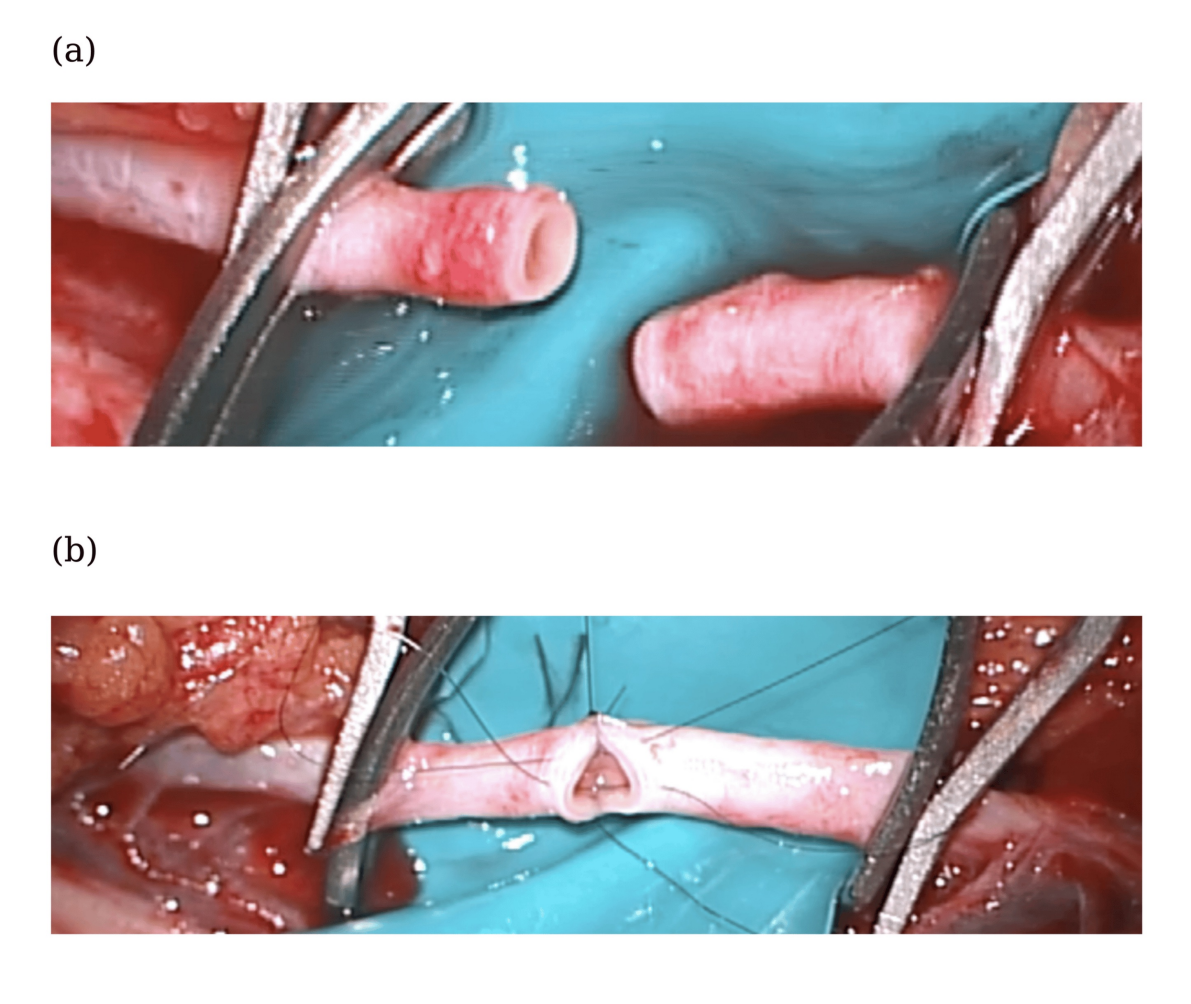
(a) Excision of the injured area. (b) End-to-end anastomosis using 8-0 nylon.

Intraoperatively, the displaced humeral fragment was found to compress the brachial artery (Fig. 2). Following careful dissection and protection of the arteries, the fracture was reduced and stabilized with one medial and two lateral Kirschner wires (K-wires) (Fig. 3). However, there was no radial pulse, and complete occlusion at the compression site was confirmed from ultrasonography. Suspecting intimal injury despite the absence of gross arterial rupture, the damaged arterial segment was resected, and end-to-end anastomosis was conducted five hours post-injury, resulting in immediate restoration of the radial pulse (Fig. 4). 

Postoperatively, the arm was immobilized with a splint. On postoperative day 7, as the surgical wound had properly healed without signs of infection and swelling of the affected limb had subsided, the splint was converted to a cast, and the patient was discharged. Seven weeks postoperatively, radiographic evaluation showed satisfactory callus formation, leading to K-wire removal. At 2 months, bone union was confirmed with a Baumann angle of 17° and a shaft-condylar angle of 47°. Five months postoperatively, the patient achieved full elbow range of motion (0° extension and 130° flexion) and normal radial artery pulsation, returning to sports activities without hindrance.

## Discussion

The treatment of this case provided us with several valuable clinical lessons. Initially, we observed that even in patients presenting with PPH, a seemingly less severe vascular presentation, rapid deterioration to significant vascular compromise is possible. This experience showed the importance of rigorous and frequent vascular assessments to ensure early detection of developing ischemia. Furthermore, intraoperative ultrasonography showed that it was useful as a rapid diagnostic tool, facilitating timely and informed decision-making during surgery. It is important to contextualize these findings with the broader understanding of supracondylar humeral fractures, which constitute 55-80% of pediatric elbow fractures and are typically managed with closed reduction with percutaneous pinning [5, 6]. However, severe fracture displacement can lead to vascular compromise and nerve injury, including PPH, a condition where the radial artery pulse is absent despite preserved peripheral perfusion. 

The optimal treatment strategy for PPH remains debated. Delniotis et al. in their systematic review advocated for initial closed reduction [7]; nevertheless, the risk of persistent ischemia, especially in cases presenting with vascular impairment, was reported in other studies. These findings show that early vascular exploration may be more appropriate than prolonged observation or repeated attempts at closed reduction in such scenarios [6, 8]. In this particular case, the patient was initially presented with PPH, rapidly developed signs of ischemia, emphasizing that vigilant vascular monitoring was necessary and the need for emergency surgical intervention preparedness. To facilitate accurate diagnosis and localization of vascular injuries, imaging modalities, including ultrasound and three-dimensional computed tomography angiography, are invaluable tools [3, 9, 10].

In previous reports, causes of persistent vascular compromise after fracture reduction were documented, including arterial spasm, constriction, bony compression, and intravascular thrombosis [4]. Our case presented a unique challenge. Despite successful brachial artery decompression, blood flow remained absent. Intraoperative ultrasound revealed complete cessation of flow at the compression site, leading to the identification of an arterial intimal injury. This enabled immediate vascular reconstruction. In this instance, intraoperative ultrasonography proved invaluable by providing a rapid and direct assessment of the distal radial artery flow and precise localization of the vascular injury. Consequently, the facilitated prompt decision-making and timely revascularization contributed to a favorable outcome without long-term complications.

## Conclusions

Supracondylar humeral fractures are a common pediatric injury; nevertheless, the risk of rapidly progressing vascular compromise necessitates diligent clinical evaluation. Clinicians should be acutely aware of potential complications and be prepared for emergent open surgery. Thus, frequent and appropriate vascular assessments are indispensable for prompt diagnosis and timely intervention, minimizing the risk of long-term sequelae. Collectively, careful monitoring and readiness for surgical exploration are essential for optimizing outcomes in these potentially high-risk injuries.

## References

[1] Skaggs DL, Flynn JM (2020). Supracondylar fractures of the distal humerus. Rockwood and Wilkin’s Fractures in Children, 9th edition.

[2] Weller A, Garg S, Larson AN (2013). Management of the pediatric pulseless supracondylar humeral fracture: is vascular exploration necessary?. J Bone Joint Surg Am.

[3] Benedetti Valentini M, Farsetti P, Martinelli O, Laurito A, Ippolito E (2013). The value of ultrasonic diagnosis in the management of vascular complications of supracondylar fractures of the humerus in children. Bone Joint J.

[4] Sato K, Tsuji H, Kurata Y (2019). Open surgical management of pediatric supracondylar humeral fracture with pulseless pink hand [article in Japanese]. Hokkaido Orthopedic Surgery of Traumatology.

[5] Griffin KJ, Walsh SR, Markar S, Tang TY, Boyle JR, Hayes PD (2008). The pink pulseless hand: a review of the literature regarding management of vascular complications of supracondylar humeral fractures in children. Eur J Vasc Endovasc Surg.

[6] Gosens T, Bongers KJ (2003). Neurovascular complications and functional outcome in displaced supracondylar fractures of the humerus in children. Injury.

[7] Delniotis I, Delniotis A, Saloupis P (2019). Management of the pediatric pulseless supracondylar humeral fracture: A systematic review and comparison study of “watchful expectancy strategy” versus surgical exploration of the brachial artery. Ann Vasc Surg.

[8] Louahem D, Cottalorda J (2016). Acute ischemia and pink pulseless hand in 68 of 404 gartland type III supracondylar humeral fractures in children: Urgent management and therapeutic consensus. Injury.

[9] Xie LW, Wang J, Deng ZQ (2021). Treatment of pediatric supracondylar humerus fractures accompanied with pink pulseless hands. BMC Musculoskelet Disord.

[10] Yamada T, Rokkaku T (2015). Ultrasonographic findings of the brachial artery in pediatric supracondylar humeral fractures [article in Japanese]. Japanese Elbow Soc.

